# The role of school functioning, physical activity, BMI, sex and age in building resilience among Ukrainian refugee children in Poland

**DOI:** 10.1038/s41598-024-55933-6

**Published:** 2024-03-04

**Authors:** Agata Korcz, Elżbieta Cieśla, Piotr Urbański

**Affiliations:** 1 Department of Didactics of Physical Activity, Poznan University of Physical Education, Krolowej Jadwigi 27/39, 61-871 Poznan, Poland; 2 Institute of Health Sciences, Jan Kochanowski University of Kielce, Żeromskiego 5, 25-369 Kielce, Poland; 3 Department of Adapted Physical Activity, Poznan University of Physical Education, Krolowej Jadwigi 27/39, 61-871 Poznan, Poland

**Keywords:** Public health, Health care

## Abstract

The study aims to examine the relationship between school functioning, physical activity (PA), sex, Body Mass Index (BMI), age, and resilience in Ukrainian children who migrated to Poland due to the war. A cross-sectional study was conducted in 2022, focusing on 248 children aged 10–15 years. The findings suggest that school environment, including enjoyment of school and strong support from teachers, plays a significant role in building resilience in children. PA enhanced the resilience of girls, whereas a higher BMI negatively impacted it. A child-friendly school environment that encourages PA and provides social support could be a promising approach for the mental health of Ukrainian refugee children.

## Introduction

Displacement and migration of children during times of war present complex and critical challenges. Conflict and instability expose children to violence, loss, and upheaval, exacerbating their vulnerabilities^1,2^. These challenges encompass disrupted lives, family separation, trauma, and limited access to essential services^3–7^. Consequently, their well-being is impacted on physiological, psychological, and social levels. While international legal and policy frameworks exist to protect them, their adequacy warrants examination. Comprehensive interventions are needed, including humanitarian assistance, education, healthcare, psychosocial support, family reunification, and long-term integration measures.

Since the outbreak of the Russo-Ukrainian war on February 24th, 2022, approximately 700,000 to 800,000 school-aged children and adolescents from Ukraine have relocated to Poland. However, many of these children have not enrolled in Polish educational institutions. Instead, they engage in distance learning offered by Ukrainian schools or have since been relocated to other countries or have returned to Ukraine. By September 2022, about 200,000 of these children had officially enrolled in Polish schools. Given the size of this group, there are growing concerns regarding the mental health of these children, the provision of appropriate conditions for them, and the effectiveness of systemic solutions.

The influx of Ukrainian children has presented several challenges reported in previous studies, including language barriers and cultural differences^8–10^. Many of these children do not speak Polish, making communicating in and adapting to a new educational system difficult. In response to these challenges, the Polish government and educational institutions have implemented programs and initiatives to support integrating Ukrainian children into Polish society^9^. These initiatives include language classes, cultural exchange programs, and counseling services, which have been recognized as effective in assisting migrant children and their families in achieving inclusion and adaptation to challenging circumstances^1,11,12^.

Nevertheless, despite these initiatives, there remains an evident gap in comprehending the full effectiveness of these measures within the school environment. Ambiguities persist regarding the impact of school stress and bullying phenomena. Language barriers, adaptation to new educational systems, and academic pressure may significantly affect the well-being of refugee children and do not facilitate their integration opportunities. Bullying is a matter of concern, intensified by the heightened vulnerability of these children. It is imperative to address these issues while also exploring other potential challenges that have not yet been extensively studied, ensuring a comprehensive approach in understanding and intervening in the school environments of refugee children.

One of the factors that plays a crucial role in mitigating the effects of war and migration, as well as facilitating adaptation in difficult circumstances for children, is resilience^5,9,13^. Some authors have offered intricate definitions that allow a multidimensional understanding of resilience. For instance, Ungar^14^ defined it as the capacity of individuals to navigate to the psychological, social, cultural, and physical resources that sustain their well-being in adverse conditions, and to negotiate for these resources to be provided in culturally meaningful ways. His approach thus considers the interactions between an individual's personal traits and the individual’s environment. Consequently, resilience in this study is understood to be particularly susceptible to contextual variation, capacity of individuals to navigate their way to the psychological, social, cultural, and physical resources that sustain their well-being. Numerous studies have demonstrated a significant correlation between resilience and social support, particularly school support, and the overall well-being of migrant children and adolescents^15,16^. Positive perceptions of the school environment have also been found to positively impact the physical, emotional, and social dimensions of health among migrant children^15,17,18^.

In addition to the factors mentioned above, other variables are considered to have both direct and indirect effects on resilience. These include psychological factors such as the level of participation, coping strategies, positive beliefs, and self-esteem, as well as physical characteristics such as PA and BMI^19,20^. Understanding the interplay between the perception of the school adjustment, school support, resilience, and health outcomes is crucial for developing effective interventions and support systems that promote the well-being of migrant children. Therefore, this study aims to investigate the association of PA, sex, BMI, age, and school functioning with resilience in children from Ukraine who migrated to Poland after the outbreak of war. We hypothesized that school environment factors (general school adjustment, social support, bullying) and biological factors (sex, age, BMI), and PA would significantly affect the resilience of Ukrainian children. The results have the potential to enhance our understanding of the relative significance of PA and school social support in improving the resilience of Ukrainian children.

## Methods

### Participants and study design

A cross-sectional research design was employed in this study to investigate the experiences of Ukrainian students who arrived in Poland following the outbreak of full-scale war. The study was conducted between September and December 2022 and involved 248 participants with an average age of 12.76 years (SD = 2.44). The sufficient sample size of 233 children was determined based on the proportion of the population, assuming a margin of error of 5%, a confidence level of 95%, and the population size (n = 4417) in the area covered by the study^21^. The survey was administered anonymously in primary schools during regular classroom time, with a Polish teacher and a Ukrainian teacher present during survey administration. Informed consent was obtained from all participants/legal guardians. The study was conducted according to the Declaration of Helsinki, with its protocol approved by the Bioethics Committee of the Poznan University of Medical Science (KB—822/22). The research team, consisting of a psychologist, an ethicist, a Polish teacher, and two Ukrainian teachers, collaborated in the development of the study to ensure its ethical considerations, cultural and context sensitivity. All research tools, including study-specific questions, were available in Ukrainian or translated into Ukrainian by two native speakers using a back-translation approach. The teachers conducting the research were trained specifically for this purpose and given guidelines to ensure optimal conditions for survey administration. These guidelines included using spacious classrooms or suitable facilities to accommodate the students. To respect the autonomy of the children, they were given the option to decline participation in the study.

### Resilience

*The Child and Youth Resilience Measure*, CYRM-R^14^, is a widely used tool for assessing social-ecological resilience in children and youth. It was developed based on data from the International Resilience Project, translated into multiple languages and demonstrated to be applicable across cultures^14^. CYMR-R, is a 5-point Likert scale measure comprising 17 positively worded items designed to assess personal and caregiver/relational resilience. Scoring entails summing the responses, with higher scores indicating greater resilience. Subscale scores can be computed for personal and caregiver/relational resilience, reflecting important relationships and individual characteristics. The measure includes specific item combinations for calculating subscale scores and provides different score ranges based on the response options. The measure’s psychometric properties have also been extensively examined and found to be suitable for use across diverse cultures worldwide^22,23^. The resilience assessment measure demonstrated high reliability with a Cronbach's α of 0.85. This tool has already been used in a group of children from Ukraine^13^.

### Functioning at school

Selected questions from the *Health Behaviour in School-aged Children* (HBSC) questionnaire were used to examine the following variables: general school adjustment, school social support (peer, classmate, and teacher support), and bullying. Six questions or measurement scales were analyzed, categorized into three or four ranges. Conventional division criteria were used, identical to the national HBSC report^24^.

In general school adjustment, the following questions were analyzed:

1. Question concerning general attitude to school: “How do you feel about school at present?” with response categories: “I like it a lot”, “I like it a bit”, “I don`t like it very much”, “I don`t like it at all”. The study analyzed answers according to three categories: “like a lot”, “like a bit”, and “don`t like”.
2. Question concerning the pressure of schoolwork (school stress): “How pressured do you feel by the schoolwork you have to do?” with answer categories: “not at all”, “a little”, “some”, “a lot”. The study analyzed answers according to three categories: “not at all”, “a little, and “some/ a lot”.

Next, in support associated with school, two scales were analyzed consisting of three statements with four response categories, ranging from “definitely disagree” to “definitely agree”. Students' responses coded on a scale ranging from 0 to 4. The obtained scores were summed to create an index representing the level of support, which ranged from 0 to 12 points. A higher score indicated a higher level of support. The score ranges were as follows: 0–5 represented a low level of support, 6–9 indicated a moderate level, and scores of 10–12, a high level of support.

Questions concerning students’ perceptions of their classmates within the school environment (e.g., “The students in my class(es) enjoy being together,” “Most of the students in my class(es) are kind and helpful,” “Other students accept me as I am”) and their perception of how their teachers felt about them (e.g., “I feel that my teachers accept me as I am”, “I feel that my teachers care about me as a person”, “I feel a lot of trust in my teachers”).

In the context of *bullying*, two questions concerning involvement as a bully or victim within the past two months were considered: Students were asked questions: “How often have you taken part in bullying another person(s) at school in the past 2 months?”; “How often have you been bullied in the past 2 months?” Both questions featured response categories ranging from “I have not bullied another person(s)/been bullied at school in the past couple of months” to “several times a week”.

Additionally, a scale consisting of four statements was used to assess perceived peer support. The statements included: “My colleagues really try to help me”, “I can count on my colleagues when things go wrong”, “I have friends and colleagues with whom I can share my joys and worries”, “I can talk to my colleagues about my problems.” For each response on this scale, students could get 1 to 7 points, and the full-scale ranges from 4 to 28 points. The higher the full score obtained, the higher the level of peer support. The reliability analysis showed a moderate to high reliability of the test assessing the level of support for the total α Cronbach = 0.81 for the individual areas of support (students: α Cronbach = 0.64; teachers 0.66, peers 0.91). An analysis of the psychometric features of the above scale indicates their univariate structure and satisfactory reliability, which was described in the technical report from the HBS study^24^.

### Physical activity

*Physical Activity Screening Measure*^25^ was used to assess PA levels. This measure quantifies the average number of days per week in which individuals engage in at least 60 min of various forms of PA. Participants subjectively report increased heart rate and experience shortness of breath (higher breath rate) during these activities. The moderate-to-vigorous physical activity (MVPA) score is calculated using the following formula: MVPA = (P1 + P2)/2, where MVPA represents the PA index, P1 indicates the number of days with PA during the past 7 days, and P2 represents the number of physically active days in a typical week. This measure has demonstrated good reliability with an intraclass correlation of 0.77^25^.

### Study-specific questions

For the analysis, the following quantitative characteristics were used: body height and weight, BMI, and PA. Additionally, the impact of family-related factors (e.g., having siblings and relatives who came to Poland, current place of residence), conflict-related experience (e.g., whether relatives remained in Ukraine, exposure to hostilities in place of residence, and experiencing the loss of a loved one in the war), intentions (e.g., the plan to return to Ukraine after the end of the war), and social support factors (e.g., the person to whom can turn for help in a difficult situation) were examined.

### Covariates

The emergence of predictors was based on a series of analyses: correlation matrix and ANOVA analyses of variance. The correlations between BMI and PA (− 0.14) and age (0.35) were statistically significant. The correlations between peers support vs. BMI, peers support vs. PA and peers support vs. age were low (− 0.05; 0.08; − 0.01 respectively) and statistically insignificant. A significant effect of classmates support and teacher support on resilience was also observed (p = 0.001; p = 0.01; p = 0.001, respectively). These variables, which were found to be significantly correlated with resilience, were selected for inclusion in the model. In addition, an analysis of the scientific literature was used in the selection of predictors. A study by Guo and Liang^26^ showed the importance of PA for resilience, so this factor was also included in the models. To avoid redundancy in the regression analysis, a tolerance index was calculated (tolerance is used as an indicator of multicollinearity). Tolerance index values in gender 1 ranged from 0.69 for the level of teacher support to 0.92 for the age variable. For gender 2 the tolerance indices ranged from 0.73 (teacher support) to 0.95 (BMI). Thus, none of the predictors found in the variables included in the regression models were redundant and highly correlated with each other.

### Statistical analysis

The distribution of quantitative variables was examined using the Shapiro–Wilk test. For height and BMI, the normal distributions were obtained (0.274; 0.058 respectively). For body mass, PA, classmate, teacher, and peer support, the distributions deviated from normal (p < 0.001); the personal resilience subscale and sum resilience for all subjects had a normal distribution (p = 0.054; p = 0.102, respectively); and the caregiver/relational subscale had a distribution deviating from normal (p < 0.001). Basic descriptive statistics such as means, standard deviations, medians, and interquartile ranges (IQR) were used to describe variables within sex groups. For categorical variables within sex groups, counts and percentages (structural indicators) were calculated. To assess the difference in quantitative variables, such as height (cm), body mass (kg), BMI (kg/m^2^), PA (hours), and peer support (points), the Student’s t-test and Mann–Whitney U test were employed (depending on normality distribution). A non-parametric chi-square test was employed to evaluate the association between sex and categorized variables. Multiple regression models were constructed to investigate the influence of factors such as PA, age, BMI, support from peers, other students, and teachers at school, and general attitude to school (liking the school) on resilience. Two models were created: a full model including all variables and a stepwise backward method to identify the most significant predictors for each sex separately. The adjusted R^2^ was used as a measure of the model's ability to explain the dependent variable.

The analyses strictly adhered to the regression model's core assumptions: linearity, independence, and absence of autocorrelation was maintained. The Shapiro–Wilk (S–W) test was utilized to assess the normality of the distribution. For the boys, the results for the first model (a multivariable model) indicated a S–W p-value of 0.868, while the second model (a stepwise regression model) showed a p-value of 0.474. For girls, the corresponding p-values were 0.630 and 0.330, respectively. Regarding autocorrelation, the Durbin-Watson d statistic was calculated, with the first model for boys indicating d = 1.733 within the range [1.557; 1.826], and the second model displaying d = 1.684 within the same range. For girls, the first model yielded d = 1.78 within the range [1.52; 1.89], and the second model resulted in d = 1.78 within the range [1.60; 1.81]. Heteroscedasticity was examined using the White test, which produced F-values with associated p-values. For the first model among boys, the result was F(p) = 0.997(0.504), and for the second model, F(p) = 0.947(0.581). Girls in the first model showed F(p) = 1.207(0.159), and in the second model, F(p) = 1.192(0.249). The Variance Inflation Factor (VIF) in the first model (boys) was 1.09 (PA) to 1.43 (support from classmates). The question “How do you feel about school at present?” yielded VIF values of 2.62 to 2.65 for the responses “Like a bit” and “Like a lot” respectively. In the second model, the lowest VIF, 1.10, was noted for medium support from teachers, and the highest VIF, 1.35, was observed for high support from classmates. Similar to the first model, the variable “How do you feel about school at present?” achieved VIF values of 2.60 and 2.54, respectively (categories: “Like a bit”; “Like a lot”).

Among the girls, the VIF values were comparable. In the first model, the range was 1.16 (support from peers) to 1.57 (high support from classmates). The second model showed VIF values ranging from 1.09 (PA) to 1.19 (high support from teachers). For the variable “How do you feel about school at present?”, the first model produced VIF values of 2.07 and 2.12, while the second model, for the categories “Like a bit” and ”Like a lot”, respectively, showed similar values.

Outlier data in the multiple regression model were identified and excluded when the absolute value of the studentized residual (SRE) was ≥ 2.5. Statistical significance was set at p < 0.05. The statistical package STATISTICA 13.3 PL was used for the analysis.

## Results

The characteristics of the study participants are presented in Table 1. Boys and girls did not exhibit significant differences in height and weight. However, BMI was higher in the girls' group than in the boys' group. The average time spent on PA was significantly higher in the boys' group than in the girls' group. There was no significant variation across sex groups for variables characterizing the respondents' family environment and willingness to return to Ukraine after the war, the presence of loved ones on the frontline, or the loss of persons due to the war (situations related to the hostilities and the declaration of return to the homeland after the end of hostilities). However, significant differences were observed in stressful situations that necessitated seeking help. Boys were more likely to seek help from another Ukrainian adult, while girls were more likely to seek help from a school counselor in difficult situations. In addition, girls were more likely to agree with the statement that their classmates try to help them and that they can talk to them about their problems. Girls also obtained a higher number of points calculated for the sum of statements concerning perceived peer support.

**Table 1:** Characteristics of Ukrainian children and adolescents according to sex.

Variables	Boys (n = 123)	Girls (n = 125)		p
	(sd)	(sd)		
Body height (cm)	154.94 (13.43)	152.31 (16.61)		0.734^A^
Body mass (kg)	44.22 (11.21)	47.35 (17.66)		0.203^A^
BMI (kg/m^2^)	18.25 (3.53)	22.04 (18.56)		**0.046**^**A**^
PA	3.74 (2.03)	2.79 (1.82)		** < 0.001**^**A**^
Siblings (n = 242) N (%)				
Only child	15 (12.61)	18 (14.63)		0.740^B^
One sibling	56 (47.06)	52 (42.28)		
Two and more	48 (40.34)	53 (43.09)		
Which of your familiars come to Poland N (%)				
Mother	114 (92.68)	113 (90.40)		0.519^B^
Father	52 (42.28)	59 (47.20)		0.436^B^
Siblings	79 (64.23)	82 (65.60)		0.821^B^
Grandparents	23 (18.70)	25 (20.00)		0.795^B^
Further family	14 (11.38)	9 (7.20)		0.256^B^
Where do you live now? (n = 247)				
In a private apartment with friends or family from Ukraine	4 (3.28)	4 (3.20)		0.394^B^
In a private apartment with friends or family from Poland	3 (2.46)	3 (2.40)		
In a rented apartment	71 (58.20)	65 (52.00)		
In a hotel or hostel	12 (9.84)	22 (17.60)		
Other place	2 (1.64)	0 (0.00)		
Do your parents plan to return to Ukraine after the end of the war? (n = 245)				
Yes	58 (48.33)	54 (43.20)		0.562^B^
No	21 (17.50)	20 (16.00)		
Hard to say	41 (34.17)	51 (40.80)		
Would you like to return to Ukraine after the war? (n = 247)				
Yes	86 (70.49)	84 (67.20)		0.568^B^
No	15 (12.30)	13 (10.40)		
Hard to say	21 (17.21)	28 (22.40)		
Did any of your relatives stay in Ukraine because of the war?				
Yes	112 (91.80)	118 (94.40)		0.420^B^
Were there any hostilities in your place of residence? (n = 244)				
Yes	78 (65.00)	67 (54.03)		0.081^B^
Have you lost a loved one in the war?				
Yes	29 (23.58)	28 (22.40)		0.826^B^
Who in Poland, apart from your family, can you turn to for help in a difficult situation?				
A school teacher	42 (34.15)	35 (28.00)		0.296^B^
A school psychologist	20 (16.26)	37 (29.60)		**0.013**^**B**^
To other adults from Ukraine	36 (29.27)	17 (13.60)		** < 0.001**^**B**^
To other adults from Poland	11 (8.94)	13 (10.40)		0.698^B^
Friend from Ukraine	33 (26.83)	33 (26.40)		0.939^B^
Friend from Poland	17 (13.82)	25 (20.00)		0.253^B^
I do not know	35 (28.46)	25 (20.00)		0.120^B^
HBSC	N (%)			
How do you feel about school at present?				
Like a lot	83 (67.48)	75 (60.00)		0.274^B^
Like a bit	29 (23.58)	31 (24.80)		
Don`t like	11 (8.94)	19 (15.20)		
How pressured do you feel by the schoolwork you have to do?				
Not at all	51 (41.46)	40 (32.00)		0.127^B^
A little	56 (45.53)	73 (58.40)		
Some or a lot	16 (13.01)	12 (9.60)		
Support from peers	18.3 3(6.53)	20.25 (6.13)		0.020^A^
Support from classmates	7.79 (2.29)	7.68 (2.19)		0.934^A^
Low	19 (15.45)	20 (16.00)		0.587^B^
Medium	77 (62.60)	84 (67.20)		
High	27 (21.95)	21 (16.80)		
Support from teachers	8.11 (2.12)	7.94 (2.12)		0.469^A^
Low	13 (10.57)	17 (13.60)		0.744^B^
Medium	84 (68.29)	81 (64.80)		
High	26 (21.14)	27 (21.60)		
A bully or victim within the past 2 months				
1 (pts)	4.88 (1.63)	5.30 (1.49)		**0.049**^**A**^
2 (pts)	4.64 (1.76)	5.06 (1.74)		0.051^A^
3 (pts)	4.84 (1.91)	5.28 (1.70)		0.075^A^
4 (pts)	3.98 (2.15)	4.61 (2.05)		**0.017**^**A**^
Sum (pts)	21.36 (6.74)	23.05 (0.92)		**0.023**^**A**^
How often have you taken part in bullying another person(s) at school in the past couple of months? (n = 246)				
I have bullied	22 (18.18)	20 (12.00)		0.175^B^
I have not bullied	99 (81.82)	105 (88.00)		
How often have you been bullied in the past couple of months?				
I have been bullied	41 (33.33)	40 (32.00)		0.823^B^
I have not been bullied	82 (66.67)	85 (68.00)		
Resilience				
Sum (pts)	60.22 (9.19)	61.42 (8.17)		0.276^C^
Personal resilience subscale	30.65 (6.50)	32.12 (5.49)		0.055^C^
Caregiver/relational resilience subscale	29.57 (4.42)	29.30 (4.57)		0.600^A^

Significant values are in bold.

^A^Mann-Whitney test.

^B^Chi square test.

^C^*t* Student test.

Tables 2 and 3 provide the results of the multiple regression analysis for both sexes. In boys, factors such as support from peers and teachers, as well as general attitude to school (liking the school), were significant factors for resilience. With an increase in peer support, the number of points awarded for stress resilience increased by 0.39 (p = 0.001). Similarly, high support from teachers, compared to low support, increased stress resilience by 2.9 (p = 0.028). Liking the school "somewhat" or "very much" compared to the category "not liking it very much" resulted in an increase in stress resilience by 2.73 points (p = 0.014) and 4.13 points (p = 0.001), respectively. The backward stepwise regression analysis confirmed the positive and significant impact of peer support (β = 0.42, p = 0.001), liking the school in both categories compared to "not liking it very much" ("somewhat liking it"—β = 2.64, p = 0.015; "liking it very much"—β = 4.31, p = 0.001), and high support from teachers compared to low support (β = 2.90, p = 0.021) on stress resilience in boys.

**Table 2:** Regression analysis on resilience with independent variables such as sex, BMI, age, PA, general attitude to school (liking the school), and support from peers, classmates, and teachers for boys.

Variables	Categories	Multivariables model				Backstep forward regression			
		Β	SE	95% CI	P	β	SE	95% CI	P
Age		− 0.28	0.27	− 0.79; 0.38	0.312				
BMI		0.14	0.20	− 0.25; 0.53	0.484				
Peer support		0.39	0.10	0.18; 0.59	** < 0.001**	0.42	0.09	0.23; 0.60	**0.001**
PA		− 0.30	0.30	− 0.98;0.30	0.322				
How do you feel about school at present?	Don’t like	Ref				Ref			
	Like a bit	2.73	1.09	− 4.90; − 0.56	**0.014**	2.64	1.07	0.53; 4.75	**0.015**
	Like a lot	4.13	1.09	2.06; 6.20	**0.001**	4.31	0.98	2.37; 6.26	**0.001**
The level of classmate support	Low level	Ref							
	Moderate level	− 1.24	0.86	− 2.94; 0.46	0.152				
	High level	0.78	1.16	− 1.52; 3.08	0.503				
The level of teacher support	Low level	Ref				Ref			
	Moderate level	− 1.15	0.92	− 2.98; 0.67	0.213	− 1.31	0.89	− 3.07; 0.44	0.141
	High level	2.90	1.30	0.31; 5.48	**0.028**	2.90	1.30	0.45; 5.35	**0.021**
R^2corrected^		**0.3821**				**0.4078**			

Significant values are in bold.

**Table 3:** Regression analysis on resilience with independent variables such as sex, BMI, age, PA, general attitude to school (liking the school), and support from peers, classmates, and teachers for girls.

Variables	Categories	Multivariables model				Backstep forward regression			
		Β	SE	95% CI	P	Β	SE	95% CI	P
Age		− 0.03	0.36	− 0.74; 0.69	0.941				
BMI		− 0.14	0.03	− 0.21; − 0.08	**0.001**	− 0.13	0.03	− 0.20; − 0.07	**0.001**
Peer support		0.08	0.11	− 0.13; 0.29	0.470				
PA		1.06	0.36	0.35; 1.76	**0.004**	0.91	0.35	0.21; 1.61	**0.011**
How do you feel about school at present?	Don`t like	Ref				Ref			
	Like a bit	− 0.82	1.05	− 2.91; 1.27	0.437				
	Like a lot	2.08	0.93	0.23; 3.93	**0.028**				
The level of classmate support	Low level	Ref							
	Moderate level	− 1.44	0.90	− 3.22; 0.34	0.112				
	High level	2.04	1.28	− 0.50; 4.59	0.114				
The level of teacher support	Low level	Ref				Ref			
	Moderate level	0.70	0.93	− 0.15; 2.55	0.751	0.40	0.92	− 1.42; 2.23	0.662
	High level	4.11	1.25	1.62; 6.59	**0.001**	6.11	1.09	3.96; 8.28	**0.001**
R^2corrected^		**0.3350**				**0.3053**			

Significant values are in bold.

In girls, high support from teachers compared to low support was also significant for resilience (β = 4.11, p = 0.001). Additionally, Ukrainian girls who "liked the school very much" compared to their peers who "didn't like it very much" scored 2.08 points higher in the test assessing their stress resilience (p = 0.028). Additionally, spending ≥ 1 h/day on PA had a positive impact on the results of the resilience (β = 1.09, p = 0.004). At the same time, higher BMI significantly reduced their resilience (β = − 0.14, p = 0.001). The backward stepwise regression confirmed the significant importance of high support from teachers (β = 6.11, p = 0.001), PA (β = 0.91, p = 0.011), and BMI (β = − 0.13, p = 0.001). The backward stepwise regression predictors explained between 30.53% (girls) and 40.78% (boys) of the total variability in resilience among Ukrainian children.

For boys, the presented multiple regression model explains 38.21% of the total variability in the outcome variable, which is the sum of points obtained in the test. The significant factors from the test results perspective are peer support (β = 0.39), liking the school in both categories: liking it very much and liking it somewhat (β = 2.73, β = 4.13), and high support from teachers (β = 2.90). All beta parameters are positive, indicating that a 1-point change on the peer support scale corresponds to a 0.39 change in the test score assessing students' resilience. The same interpretation applies to liking the school. Children who somewhat like the school than those who dislike it achieve a 2.73-point difference in the test. Furthermore, children who like the school very much compared to those who somewhat dislike it achieve an even greater difference (4.13 points). High support from teachers positively affects the test score by 2.90 points. The backward stepwise analysis confirmed the significance of these factors.

## Discussion

This study examines the relationships between the school environment—particularly general attitude to school, school social support and bullying, PA, sex, age, BMI, and resilience, among Ukrainian children who migrated to Poland following the onset of the Russo-Ukrainian war on February 24th, 2022. Our findings support the hypothesis that school functioning, sex, BMI, and PA would significantly affect the resilience of Ukrainian children. A positive influence of a high level of teacher support and positive school experience on resilience in both boys and girls were observed. These results are consistent with previous research, consistently demonstrating the beneficial effects of social support on resilience^27–29^.

The migration of children from a war-affected country to an unfamiliar environment involves leaving behind familiar surroundings, including their previous schools and close relationships. Despite the digitalization of social life and shift to new educational environments, Ukrainian children may face challenges in forming or maintaining meaningful peer relationships. The absence of their usual social networks may pose challenges for the social integration and emotional wellbeing. Establishing new social bonds becomes crucial for these young individuals. In addition, a recent study by Urbanski et al.^13^ highlighted the key role of relational resilience in shaping children's mental health in challenging circumstances, particularly when they experience loss and conflict-related trauma.

A significant impact of peer support on resilience was observed, specifically among boys, which is consistent with research by Yearwood et al.^30^, where additionally higher levels of peer support were associated with a decreased impact of trauma on internalizing and externalizing symptoms. Other studies, such as Bokhorst, et al.^31^ among 9–18-year-old students, have indicated that girls perceived more support from teachers, classmates, and friends than boys. The discrepancy with our study could be attributed to the unique circumstances Ukrainian children face. These children, having fled war in Ukraine in 2022 and resettled in Poland, may exhibit different patterns of seeking and perceiving support due to the traumas and upheavals they experienced. Such intense experiences can significantly influence how children internalize support structures, potentially making boys in our study more reliant on peer support for resilience, as opposed to the general population studied by Bokhorst et al.^31^. Girls tend to take a more proactive approach in seeking professional and community assistance compared to boys. They generally feel that their classmates would provide them with support and are more comfortable discussing their issues with peers, which contributes to a higher perception of peer support. On the other hand, boys tend to seek help more from Ukrainian adults, in contrast to girls who are inclined to approach a school counselor in challenging situations.

Our research also identified additional sex-related differences in PA levels, aligning with findings from other studies^31–34^. Boys generally tend to engage in more PA than girls, which may contribute to differences in resilience. Some studies have suggested that girls may be more susceptible to stress related to social relationships and social pressure^35,36^. Anniko et al.^37^ highlighted that school was the most common source of stress across all time points of study, with girls reporting considerably more stress than boys. This may influence their willingness to participate in PA. Therefore, it must be considered that girls often experience heightened sensitivity towards their bodies and concerns about their appearance, which can affect their stress levels and willingness to engage in activities, including PA. Girls who have moved to Poland from Ukraine are confronted with a new situation, facing new peers and a new environment. They might experience a certain degree of comparison or competition with Polish girls, potentially making it more difficult for them to adapt to the new situation. Nevertheless, this problem requires further research.

The current study revealed PA’s significance for resilience among girls which aligns with a growing body of research that supplies evidence for a positive association between PA and mental health outcomes in children^19,38,39^. Moljord et al.^40^ pointed out that higher levels of PA were associated with lower levels of depressive symptoms for girls, this association was not observed among boys. Ho et al.^19^ showed that the indirect associations between PA level and mental well-being were comparable in female and male students. Still, the direct association was much stronger in females than in males. It is also likely that the combination of reduced PA and war-related stress catalyzes the interdependence of these variables in girls. The relationship between PA and resilience may be complex. It could be influenced by several factors, such as cultural differences between populations, which might lead to different sex patterns in participation in certain PA.

When examining the impact of BMI on adolescent resilience, recent studies shed light on this significant relationship. Research by Wardle et al.^41^ demonstrated that in adolescents, depressive symptoms are not significantly more prevalent in obese individuals compared to their normal-weight peers, regardless of sex. However, Borinsky et al.^42^ revealed that body size dissatisfaction was associated with low resilience in adolescents. Additionally, other research indicates that being overweight is associated with psychological distress in adolescent girls but not boys^43^. These findings are consistent with those of our study, which suggests that BMI has a significant negative impact on the resilience of girls. Given the context of these studies, especially considering that their participants were assessed during a war—a period characterized by heightened psychological tension and stressors—this relationship appears to be justified. However, to substantiate this association, a longitudinal analysis comparing BMI, PA and resilience is imperative.

In addition, our study found that more girls than boys were involved in bullying, both as victims and perpetrators. It has been shown in previous studies that social support is a protective factor among adolescents in the context of bullying [48], with those not involved in bullying reporting the highest levels of peer and maternal support. The significant interaction between the groups of students involved in bullying and peer social support underscores the importance of building positive interpersonal relationships in reducing the impact of bullying on mental well-being [49]. It was also noted that school bullying is a risk factor for reduced PA [50]. Given the negative consequences of being bullied and engaging in bullying, it is essential to take measures to minimize this phenomenon.

In conclusion, the observed differences between girls and boys could be attributed to various potential factors. Firstly, these differences might be sex-specific responses to the traumas of war, such as varying levels of sensitivity, cultural norms, and distinct communication styles, for example, boys may display characteristics of bravery and valor, as culturally or socially expected, masking their true emotional vulnerabilities. Alternatively, it could reflect the educators' preparedness, suggesting that teachers or caregivers were not adequately trained to address the sex-specific needs and vulnerabilities. Over time, it would be intriguing to monitor how these patterns evolve.

Importantly, while adolescents generally perceived more support from their peers compared to other sources of social support, boys reported higher levels of peer support than girls^44^. These patterns, confirmed in our studies, suggest that support-seeking behaviors and perceptions of support vary across sexes, indicating that interventions and support systems must be tailored to address the unique needs and preferences of boys and girls. Girls usually scored higher in seeking social support and problem-solving among adolescent, while boys scored higher in avoidant coping^45^. Therefore, encouraging adaptive coping strategies, such as seeking social support and problem-solving, may be particularly beneficial for enhancing resilience in this group. Individual close friendships were identified as an important potential protective mechanism accessible to most adolescents^46^.

### Study limitations

Firstly, the reliance on self-reported data introduces the possibility of response biases or inaccuracies. Secondly, despite efforts to ensure confidentiality and optimal conditions for questionnaire completion, it cannot be definitively guaranteed that students did not communicate or influence each other during data collection. Thirdly, while our study aimed to examine the impact of PA, sex, BMI, age, and school functioning on resilience, there may be other relevant factors that were not considered in this research. A further limitation arises from the varied backgrounds of the Ukrainian children, their differing arrival times across various cities leading to a wide range of war experiences and effects. The study's inability to specifically evaluate the impact of wartime conditions leaves this aspect unexplored. Lastly, despite the previously assessed reliability of tools used in various settings, as well as their utilization among Ukrainian children and consultations with native speakers and specialists from different areas, the unique context of war necessitates deep consideration of these tools in future studies.

### Implications for future studies

This study emphasizes the importance of long-term research into the resilience of Ukrainian children who have relocated to Poland following the outbreak of war. The focus should be on examining the quality of these children's functioning within Polish school environments, particularly how their sex-specific needs and challenges can be effectively addressed. A vital area of concentration includes preparing specialists, teachers, caregivers, educators, and psychologists, as well as equipping peers to facilitate the inclusion of Ukrainian children within the Polish school environment. Potential initiatives to ease this process could involve programs aimed at increasing participation in PA, along with mentoring and counseling programs. These approaches could play a significant role in enhancing the adaptation and integration of Ukrainian children into their new educational and social settings. In addition, given the extensive time children spend online and the abundance of content related to migration, war, and societal dichotomies, it also seems essential to examine the issue of cyberbullying.

## Conclusions

The study highlights the impact of sex, school functioning, PA, and BMI on the resilience of Ukrainian children who migrated to Poland due to the Russo-Ukrainian war. Key findings include the positive influence of teacher support and positive school experiences on resilience for both boys and girls. For boys, peer support and liking school significantly impacted resilience, while for girls, higher teacher support and PA were crucial, with higher BMI negatively affecting their resilience. The study underscores the need for tailored support systems in schools, considering the unique needs of boys and girls in coping with stress and trauma.

## Data Availability

The datasets used and analyzed in the current study are available from the corresponding author upon reasonable request.
